# Relationship between the Responsiveness of Amyloid β Protein to Platelet Activation by TRAP Stimulation and Brain Atrophy in Patients with Diabetes Mellitus

**DOI:** 10.3390/ijms232214100

**Published:** 2022-11-15

**Authors:** Takamitsu Hori, Daisuke Mizutani, Takashi Onuma, Yu Okada, Kumi Kojima, Tomoaki Doi, Yukiko Enomoto, Hiroki Iida, Shinji Ogura, Takashi Sakurai, Toru Iwama, Osamu Kozawa, Haruhiko Tokuda

**Affiliations:** 1 Department of Neurosurgery, Gifu University Graduate School of Medicine, Gifu 501-1194, Japan; 2 Department of Pharmacology, Gifu University Graduate School of Medicine, Gifu 501-1194, Japan; 3 Department of Metabolic Research, Research Institute, National Center for Geriatrics and Gerontology, Obu 474-8511, Japan; 4 Department of Neurosurgery, Toki Municipal General Hospital, Toki 509-5193, Japan; 5 Department of Anesthesiology and Pain Medicine, Gifu University Graduate School of Medicine, Gifu 501-1194, Japan; 6 Department of Emergency and Disaster Medicine, Gifu University Graduate School of Medicine, Gifu 501-1194, Japan; 7 Center for Comprehensive Care and Research on Memory Disorders, National Center for Geriatrics and Gerontology, Obu 474-8511, Japan; 8 Department of Cognitive and Behavioral Science, Nagoya University Graduate School of Medicine, Nagoya 466-8550, Japan; 9 Department of Clinical Laboratory/Medical Genome Center, National Center for Geriatrics and Gerontology, Obu 474-8511, Japan

**Keywords:** Amyloid β protein, platelet, thrombin receptor-activating protein, platelet-derived growth factor, diabetes mellitus, brain atrophy

## Abstract

Type 2 DM is a risk factor for dementia, including Alzheimer’s disease (AD), and is associated with brain atrophy. Amyloid β protein (Aβ) deposition in the brain parenchyma is implicated in the neurodegeneration that occurs in AD. Platelets, known as abundant storage of Aβ, are recognized to play important roles in the onset and progression of AD. We recently showed that Aβ negatively regulates platelet activation induced by thrombin receptor-activating protein (TRAP) in healthy people. In the present study, we investigated the effects of Aβ on the TRAP-stimulated platelet activation in DM patients, and the relationship between the individual responsiveness to Aβ and quantitative findings of MRI, the volume of white matter hyperintensity (WMH)/intracranial volume (IC) and the volume of parenchyma (PAR)/IC. In some DM patients, Aβ reduced platelet aggregation induced by TRAP, while in others it was unchanged or rather enhanced. The TRAP-induced levels of phosphorylated-Akt and phosphorylated-HSP27, the levels of PDGF-AB and the released phosphorylated-HSP27 correlated with the degree of platelet aggregability. The individual levels of not WMH/IC but PAR/IC was correlated with those of TRAP-stimulated PDGF-AB release. Collectively, our results suggest that the reactivity of TRAP-stimulated platelet activation to Aβ differs in DM patients from healthy people. The anti-suppressive feature of platelet activation to Aβ might be protective for brain atrophy in DM patients.

## 1. Introduction

Type 2 diabetes mellitus (DM) has been recently known to be associated with neurodegenerative and cerebrovascular diseases [1]. DM is a significant risk factor for all-cause dementia, Alzheimer’s disease (AD), and vascular dementia [2]. The underlying mechanism of the association between dementia and DM has not yet been fully elucidated; however, clinical features such as cognitive deficits, large-and small-vessel disease, cerebral atrophy, hypometabolism, and impaired insulin activation in the brain, overlap between type 2 DM and AD [1].

Amyloid β protein (Aβ) is a fragment of amyloid precursor protein (APP) [3,4]. APP is a type 1 transmembrane glycoprotein and is expressed and processed by two alternative pathways, one is the amyloidogenic and the other is the non-amyloidogenic pathway [3,5]. APP is cleaved first by α-secretase (non-amyloidogenic pathway) or β-secretase (amyloidogenic pathway). In the amyloidogenic pathway, γ-secretase subsequently produces the cleavage fragments 40-amino acid (Aβ1–40) and 42-amino acid (Aβ1–42) [6]. AD is the most common type of dementia, accounting for 50–56% of cases [7]. The deposition of Aβ in the brain parenchyma is well known to be implicated in the neurodegeneration that occurs in AD [8]. APP is abundant in the megakaryocytes and human platelets [9]. Human platelets produce APP metabolites and store APP and Aβ in α-granules, which are released into the blood plasma on the platelet degranulation [9,10]. Since most Aβ is derived from platelets, platelets are attracting attention as a new therapeutic target for AD [11]. Regarding the effect of Aβ on platelet functions, we have recently shown that Aβ negatively regulates human platelet activation induced by thrombin stimulation, in healthy people [12].

Human platelets play an important role in primary hemostasis and pathological thrombus formation [13]. Platelet activation is triggered by the initial tethering of platelets to the injured vessels [13]. Activated human platelets secrete autocrine/paracrine mediators such as adenosine diphosphate (ADP), release thromboxane A2 and promote the repairment of vascular injury [13]. Additionally, mitogenic mediators such as platelet-derived growth factor-AB (PDGF-AB) are secreted from activated platelets as granule contents, which mainly act on connective tissues, including vascular endothelial/smooth muscle cells [13]. Thrombin, a serine protease rapidly generated from circulating prothrombin at the injured site, is not only a coagulation factor but also a direct activator of platelets [14]. Thrombin binds to protease-activated receptors (PARs) on the surface of platelets and cleaves their amino-terminal exodomain to unmask a new amino terminus [13]. PARs belong to the GTP-binding protein-coupled receptor superfamily. It is currently known that two classes of PARs, PAR1 and PAR4, are expressed on human platelets, and PAR1 mainly contributes to their activation [13]. Thrombin receptor-activating protein (TRAP), a 14-amino acid peptide identical sequence to the new amino-terminus derived from the thrombin-mediated cleavage, is a potent thrombin receptor activator [15]. We have previously reported that platelets stimulated by TRAP lead to the secretion of PDGF-AB [16] and that TRAP-induced activation of Akt in addition to p38 MAP kinase positively regulates the release of phosphorylated heat shock protein 27 (HSP27) from human platelets in patients with DM [17].

Magnetic resonance (MR) imaging is widely accepted as a useful tool for the assessment of intracranial damages associated with dementia. White matter hyperintensity (WMH), which presents a high signal area in T2-weighted and fluid-attenuated inversion recovery (FLAIR) images and isointense or low signal area in T1-weighted images, has been proposed to play an important role in geriatric syndromes including cognitive impairment in the elderly [18,19,20,21]. In DM patients, it has been reported that increased volumes of WMH in comparison with non-diabetic patients [22,23]. In addition, deteriorated brain atrophy has been observed in patients with AD and type 2 DM [24], and postprandial hyperglycemia is recently supposed to be associated with brain atrophy as well as WMH in elderly patients with type 2 DM [25]. Given the fact that platelets are recognized to play important roles in the onset and progression of AD as abundant storage of Aβ [10], the changes in the reactivity of platelets in DM patients are speculated to affect these findings of MR imaging. In this study, we investigated the effects of Aβ on the TRAP-stimulated platelet activation in patients with DM, and the relationship between the responsiveness to Aβ and the quantitative findings of MR imaging.

## 2. Results

### 2.1. Characterization of the Subjects

The clinical and biochemical characteristics of the subjects (n = 96) are presented in Table 1. The HbA1c levels of the subjects were 8.5 (7.4–9.8)%, those were significantly higher than the upper limit value of the normal range (5.9%). The anthropometrical indexes were within the normal ranges in Japanese, and there was no significant change in metabolic variables.

### 2.2. The Effect of Aβ to Platelet Activation by TRAP Stimulation in Patients with Diabetes Mellitus (DM)

**Figure 1:**
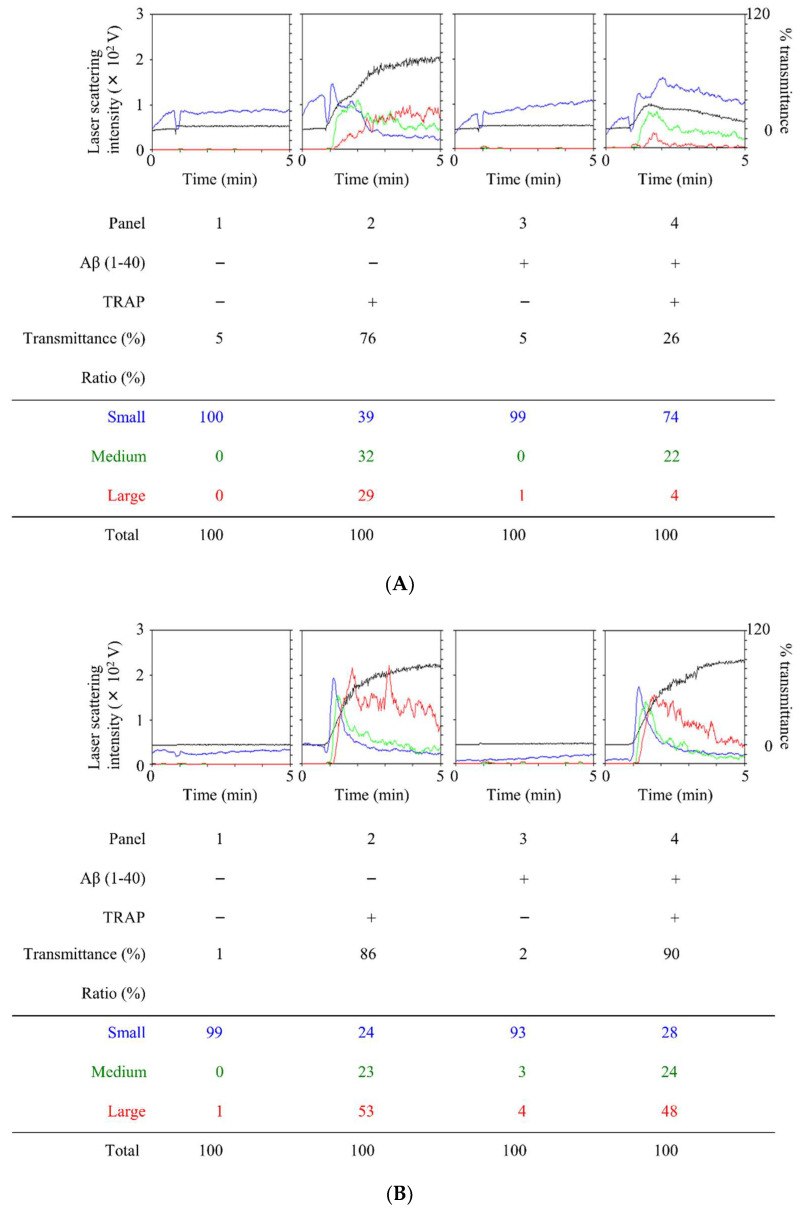
Effect of amyloid β protein (Aβ) (1–40) on thrombin receptor-activating protein (TRAP)-stimulated aggregation of human platelets in diabetes mellitus (DM) patients. PRP from DM patients was pretreated with 10 μM of Aβ (1–40) or vehicle at 37 °C for 15 min and then stimulated with 8–15 μM of TRAP or vehicle for 5 min. The dose of TRAP achieving a transmittance of 70–100% recorded using a PA-200 aggregometer (PA-200 apparatus; Kowa Co., Ltd., Tokyo, Japan) was adjusted individually. The black line indicates the percentage of transmittance of each sample (isolated platelets recorded as 0%, and PPP recorded as 100%). The blue line indicates small aggregates (9–25 μm), the green line indicates medium aggregates (25–50 μm), and the red line indicates large aggregates (50–70 μm). The lower panel presents the distribution (%) of aggregated particle size, measured by laser scattering. The representative results of two different responsiveness to Aβ, suppressed (**A**) and unchanged (**B**), are presented, respectively.

It has been reported that Aβ can potentiate human platelet aggregation [26,27,28]. However, our previous study clearly demonstrated that Aβ decreases platelet aggregation induced by TRAP stimulation in healthy adults [12]. Therefore, we first investigated the effect of Aβ on platelet activation by TRAP stimulation in patients with DM. In our experimental conditions, Aβ did not induce platelet aggregation. In some individuals, TRAP-stimulated platelet aggregation, detected by the increase of transmittance and an increased ratio of large aggregates with a decreased ratio of small aggregates, was suppressed by 15 min of Aβ pretreatment, as in healthy adults (Figure 1A). On the other hand, in the others, unlike healthy adults, the platelet aggregation response was unchanged or enhanced (Figure 1B). 

### 2.3. Effect of Aβ on TRAP-Induced Phosphorylation of Akt and HSP27 in Human Platelets of DM Patients

**Figure 2:**
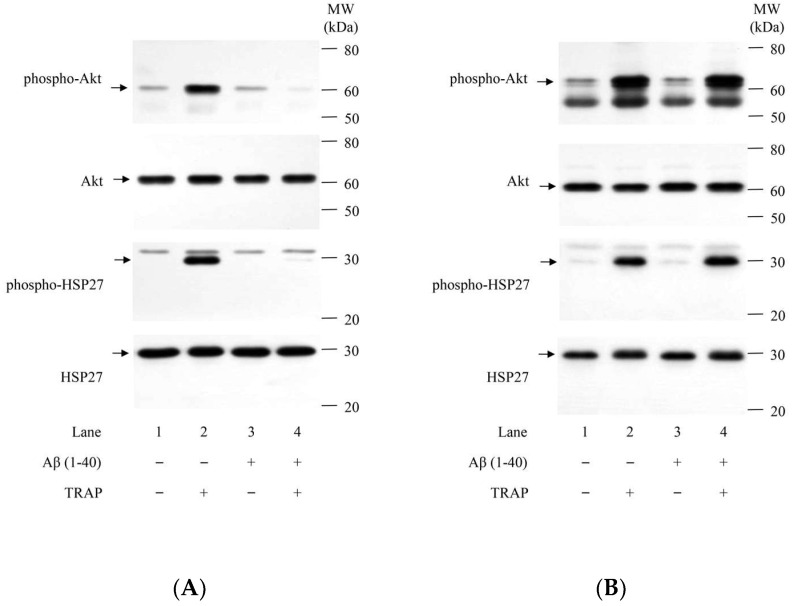
Effects of Aβ (1–40) on TRAP-induced phosphorylation of Akt, and HSP27 in human platelets of DM patients. PRP was pretreated with 10 μM of Aβ (1–40) or vehicle at 37 °C for 15 min and then stimulated with 8–15 μM of TRAP or vehicle for 5 min. The dose of TRAP achieving a transmittance of 70–100% recorded using a PA-200 aggregometer was adjusted individually. The reaction was terminated by the addition of an ice-cold EDTA solution. The lysed platelets were then subjected to Western blot analysis using antibodies against phospho-specific Akt (Thr-308) and Akt and phospho-specific HSP27 (Ser-78) and HSP27. The representative results of two different responsiveness to Aβ, suppressed (**A**) and unchanged (**B**), are presented, respectively. The data from identical samples of Figure 1 are shown. Phospho-specific Akt (Thr-308) antibodies and Akt antibodies were purchased from Cell Signaling Technology, Inc. (Beverly, MA, USA). Phospho-specific HSP27 (Ser-78) antibodies was purchased from Enzo Life Science, Inc. (Plymouth Meeting, PA, USA). HSP27 antibodies were purchased from Santa Cruz Biotechnology, Inc. (Santa Cruz, CA, USA).

We have previously shown that TRAP-induced phosphorylation of p38 MAP kinase, but not JNK, is followed by the phosphorylation of HSP27, leading to the release of phosphorylated HSP27 into the plasma [16] and that Akt in addition to p38 MAP kinase positively regulates the TRAP-induced release of phosphorylated HSP27 from human platelets in patients with DM [17]. Thus, we examined the effect of Aβ on TRAP-induced phosphorylation of Akt and HSP27 in human platelets. Aβ at a dose of 10 μM, which by itself barely affected the phosphorylation of Akt and HSP27, significantly decreased the levels of TRAP-induced phosphorylation of Akt and HSP27 (Figure 2A). The suppressive effect of Aβ on the phosphorylation of Akt and HSP27 is observed in the case where the platelet aggregation is suppressed (Figure 1A and Figure 2A were derived from the same samples). On the other hand, there are some cases in which Aβ did not suppress the levels of TRAP-induced phosphorylation of Akt nor HSP27 (Figure 2B). In these cases, TRAP-induced platelet aggregation was not suppressed by Aβ (Figure 1B and Figure 2B were derived from the same samples).

### 2.4. Effects of Aβ on TRAP-Induced Secretion of PDGF-AB and Release of Phosphorylated HSP27 from Human Platelets of DM Patients

**Figure 3:**
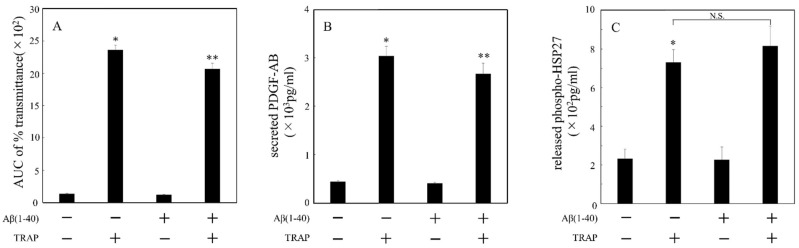
Effects of Aβ (1–40) on the TRAP-induced secretion of PDGF-AB and release of phosphorylated HSP27 from human platelets of DM patients. PRP was pretreated with 10 μM of Aβ (1–40) or vehicle at 37 °C for 15min and then stimulated with 8–15 μM of TRAP or vehicle for 5 min. The dose of TRAP achieving a transmittance of 70–100% recorded using a PA-200 aggregometer was adjusted individually. The reaction was terminated by the addition of an ice-cold EDTA solution. Platelet aggregation determined by PA-200 aggregometer and presented as AUC of % transmittance (**A**). The mixture was centrifuged at 10,000× *g* at 4 °C for 2 min, and the supernatant was subjected to ELISA for PDGF-AB (**B**) and phosphorylated HSP27 (**C**). The results obtained from 96 DM patients are shown. Each value represents the mean ± standard error of the mean. * *p* < 0.05, compared to the value of control. ** *p* < 0.05, compared to the value of agonist alone. N.S. indicates not significant.

Previous studies from our laboratory have demonstrated that human platelets activated by TRAP lead to the secretion of PDGF-AB and release of phosphorylated HSP27 [16]. We examined the effect of Aβ on the TRAP-induced PDGF-AB secretion and phosphorylated HSP27 release, as well as the area under the curve (AUC) of the light transmittance obtained from aggregometer. Aβ at a dose of 10 μM decreased AUC of % transmittance in platelet aggregation stimulated by TRAP (Figure 3A). Aβ at a dose of 10 μM decreased TRAP-induced secretion of PDGF-AB in patients with DM, but the reduction was only about 10% (Figure 3B). In contrast, Aβ at a dose of 10 μM did not affect the TRAP-induced release of phosphorylated HSP27 (Figure 3C). From these findings, an approach to individual Aβ-responsiveness should be required to evaluate the Aβ action.

### 2.5. The Relationship in the Aβ-Effects between Aggregation and Secreted PDGF-AB or Released Phosphorylated HSP27 in TRAP-Stimulated Human Platelets of DM Patients

**Figure 4:**
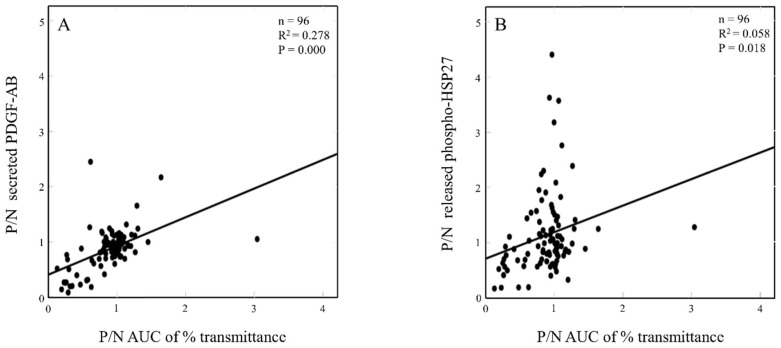
The relationship in the individual Aβ-effects between aggregation and secreted PDGF-AB or released phosphorylated HSP27 in TRAP-stimulated human platelets of DM patients. We indicated individual Aβ-effect on the TRAP-stimulated platelets of each parameter as a calculated ratio, Aβ-positive/Aβ-negative (P/N) and plotted the individual P/N AUC of % transmittance in platelet aggregation against P/N secreted PDGF-AB (**A**) or P/N released phosphorylated HSP27 (**B**) induced by TRAP stimulation in the platelets of type 2 DM patients, respectively. The data from 96 subjects were plotted and analyzed by linear regression analysis.

To illustrate the individual responsiveness to Aβ, we calculated a ratio of Aβ-effect as the value obtained from the platelets pretreated with Aβ/the value without Aβ and indicated as Aβ-positive/Aβ-negative (P/N) in each parameter. To clarify whether the effect of Aβ on TRAP-induced platelet aggregation is related to Aβ-effect on the secretion of PDGF-AB or the release of phosphorylated HSP27 from TRAP-stimulated platelets, we plotted the individual P/N AUC of % transmittance in platelet aggregation against P/N secreted PDGF-AB or P/N released phosphorylated HSP27 induced by TRAP stimulation in the platelets of type 2 DM patients, respectively. P/N AUC of % transmittance was significantly correlated with P/N secreted PDGF-AB (R^2^ = 0.278, *p* = 0.000) (Figure 4A), and significantly correlated with P/N released phosphorylated HSP27 (R^2^ = 0.058, *p* = 0.018) (Figure 4B), respectively.

### 2.6. The Relationship between MR Imaging Findings and Aβ-Effects on the Aggregation, Secreted PDGF-AB and Released Phosphorylated HSP27 in TRAP-Stimulated Human Platelets of DM Patients

**Figure 5:**
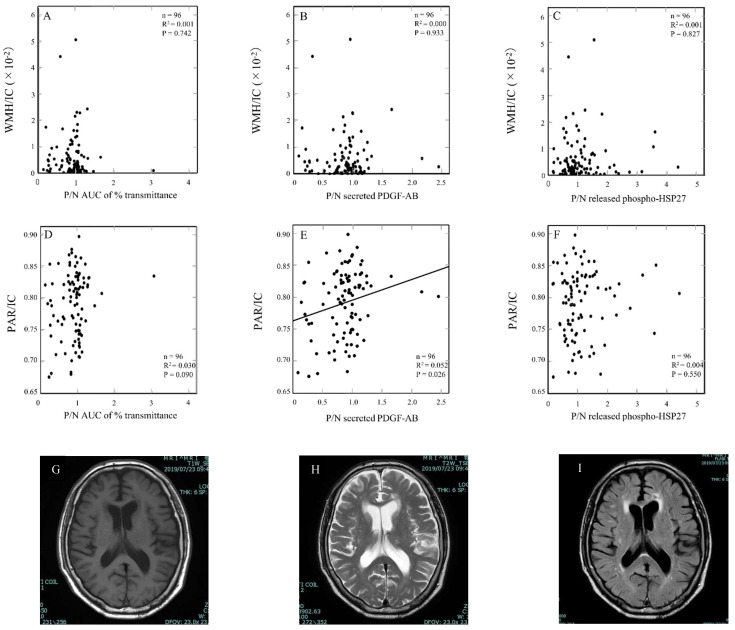
The relationship between individual levels of magnetic resonance (MR) imaging findings and Aβ-effects on the aggregation, secreted PDGF-AB and released phosphorylated HSP27 in TRAP-stimulated human platelets of DM patients. White matter hyperintensity (WMH) and brain atrophy in MR imaging were analyzed individually by an automatic segmentation application (SNIPER). Global brain atrophy was calculated as the parenchyma (PAR) volume. WMH and PAR were divided by the intracranial (IC) volume to adjust for the size of each patient’s brain. (**A**–**C**) WMH/IC were plotted against P/N AUC of % transmittance (**A**), P/N secreted PDGF-AB (**B**), P/N released phosphorylated HSP27 (**C**). (**D**–**F**) PAR/IC were plotted against P/N AUC of % transmittance (**D**), P/N secreted PDGF-AB (**E**), P/N released phosphorylated HSP27 (**F**). These data were plotted and analyzed by linear regression analysis. Representative MR images with 1.5T MR scanners are shown; T1-weighted (**G**), T2-weighted (**H**) and FLAIR (**I**) MR imaging.

Several studies have reported that WMH volume is increased in patients with DM compared with non-diabetic subjects [22,23]. Postprandial hyperglycemia is closely related to deteriorating WMH and brain atrophy in older patients with type 2 DM [25]. In addition, it is known that platelet function is altered in DM patients [17]. Thus, we investigated the relationship between WMH and Aβ-effects on TRAP-stimulated platelet activation. We plotted WMH/IC against P/N AUC of % transmittance, secreted PDGF-AB, and released phosphorylated HSP27. There were no significant relationships between WMH/IC and P/N AUC of % transmittance (R^2^ = 0.001, *p* = 0.742) (Figure 5A), P/N secreted PDGF-AB (R^2^ = 0.000, *p* = 0.933) (Figure 5B), P/N released phosphorylated HSP27 (R^2^ = 0.001, *p* = 0.827) (Figure 5C).

We next investigated the relationship between brain atrophy and Aβ-effects on TRAP-stimulated platelet activation. We plotted PAR/IC against P/N of AUC % transmittance, secreted PDGF-AB and released phosphorylated HSP27. Although it was not significant, there was a tendency of correlation between PAR/IC and P/N of AUC % transmittance (R^2^ = 0.030, *p* = 0.090) (Figure 5D). PAR/IC was significantly correlated with P/N secreted PDGF-AB (R^2^ = 0.052, *p* = 0.026) (Figure 5E). However, there was not any correlation between PAR/IC and P/N released phosphorylated HSP27 (R^2^ = 0.004, *p* = 0.550) (Figure 5F).

## 3. Discussion

In the present study, we investigated the effects of Aβ on the TRAP-stimulated platelet activation in patients with DM, and the relationships between the responsiveness of platelets to Aβ and WMH or brain atrophy. First of all, we examined the effect of Aβ on TRAP-induced platelets activation in type 2 DM patients and found that Aβ suppressed TRAP-induced platelet aggregation in some cases as shown in healthy people [12], while in others, the aggregation was unchanged or rather enhanced. In addition, in the cases where platelet aggregation is suppressed by Aβ, the effect seems to be milder than that observed in our previous report conducted in healthy volunteers [12]. Although there are some conflicting data in regard to the modulation of platelet function from Aβ in healthy subjects [26,27,28], our present findings suggest that the effect of Aβ on platelet aggregation is not mainly suppressive as previously shown in healthy subjects but rather heterotypic in DM subjects. We next examined the TRAP-induced phosphorylation levels of Akt and HSP27 under Aβ pretreatment and found that the TRAP-induced phosphorylation levels of Akt and HSP27 varied among diabetic patients, however, those of Akt and HSP27 were linked with the levels of platelet aggregation. It is likely that the reactivity of platelets to the TRAP stimulation under Aβ alters in DM patients compared with healthy people. In our previous reports [16,17], TRAP-stimulated platelets secrete PDGF-AB and release phosphorylated HSP27 into plasma accompanying its activation. Therefore, we further investigated the effect of Aβ on TRAP-induced PDGF-AB secretion and phosphorylated HSP27 release in patients with DM. We found that Aβ significantly attenuates PDGF-AB secretion induced by TRAP in patients with DM but the degree of reduction is much milder than that in healthy people. Regarding the phosphorylated HSP27 release, Aβ did not affect the phosphorylated HSP27 release. It is probable that these results may reflect the different reactivity of the platelet to the TRAP stimulation among individuals, including the enhanced, reduced and unchanged platelet aggregation compared with TRAP stimulation alone. To clarify the platelet reactivity to the TRAP stimulation under the existence of Aβ, we further examined the relationship between the % transmittance of AUC and secreted PDGF-AB or released phosphorylated HSP27. We found a positive correlation between the % transmittance of AUC and secreted PDGF-AB, and the % transmittance of AUC and released phosphorylated HSP27. Although an increased ratio of large aggregates with a decreased ratio of small aggregates indicates an advance of platelet aggregation highly qualitatively; however, the data by themselves were not quantitative and inadequate to search for correlations under statistical analyses. Taken together, it is most likely that the reactivity of platelets to the TRAP stimulation under Aβ alters in the DM patients compared with healthy people and the altered reactivity is not uniform among DM patients, which could be detected by the changes in platelet aggregation, PDGF-AB secretion and phosphorylated HSP27 release. Using these parameters which indicate the changes in platelet reactivity, we further investigated the roles of platelets in the WMH and brain atrophy.

WMH is known as one of the cerebral microvascular diseases. WMH is a common finding in the aged brain and several studies have reported increased volumes of WMH in DM patients compared to non-diabetic patients [22,23]. Thus, we investigated the relationship between WMH and TRAP-induced platelet aggregation pretreated by Aβ. The relationships between WMH and AUC of the % transmittance, secreted PDGF-AB, and released phosphorylated HSP27 were examined, but no correlations were found.

Accumulating evidence indicates that diabetes is associated with global and regional brain atrophy [23,29,30,31,32]. Thus, we next investigated the relationship between brain atrophy and TRAP-induced platelet aggregation and found that PAR/IC was correlated with secreted PDGF-AB, and there was a tendency of correlation between PAR/IC and AUC of % transmittance. To the best of our knowledge, this study was the first to find a link between brain atrophy and Aβ action on TRAP-stimulated platelet activation. Our results indicated that DM patients whose platelet reactivity to the TRAP stimulation are enhanced by Aβ are likely to have less brain atrophy. In our present study, we have already shown that the platelet reactivity alters in DM patients, but the changes are not uniform among DM patients. Given the fact that PDGF-AB are secreted from activated platelets’ granules, enhanced platelet reactivity to the TRAP stimulation under the existence of Aβ, in turn, an anti-suppressive feature of platelet activation to Aβ, may be protective against brain atrophy, leading that some “anti-atrophic factor” might be released from activated platelets beyond Aβ-abundant condition at least in DM patients.

In general, it is well known that platelet function is altered in DM patients, and reported that platelet hyperreactivity has been observed in DM patients, which contributes to the enhanced risk of atherothrombotic events [33,34]. In addition, it has been suggested that platelet activation observed in DM patients may contribute to the development of AD [35]. In the present study, we also observed changes in platelet function in DM patients, and these changes are not uniform among DM patients. It is probable that the diversity in the altered platelet function could reflect the timing of the onset or progression of cognitive dysfunction diseases including AD.

The limitation of the present study is the lack of screening for cognitive function such as Mini-Mental State Examination (MMSE) or Revised Hasegawa’s Dementia Scale (HDS-R) when platelet function was assessed. Another limitation is that the findings are based on in vitro experiments that do not take into account in vivo disease conditions, such as blood-brain barrier leaks, vascular wall damage, or underlying microbleeds. Therefore, further investigation is needed to elucidate the exact mechanism underlying the changes in platelet function and its effect on cognitive dysfunction disease. In addition, studies including younger subjects and selected participants with similar disease stages would be essential for the next step.

## 4. Materials and Methods

### 4.1. Materials

Aβ peptide (Human, 1–40) (Asp-Ala-Glu-Phe-Arg-His-Asp-Ser-Gly-Tyr-Glu-Val-His-His-Gln-Lys-Leu-Val-Phe-Phe-Ala-Glu-Asp-Val-Gly-Ser-Asn-Lys-Gly-Ala-Ile-Ile-Gly-Leu-Met-Val-Gly-Gly-Val-Val trifluoroacetate form), was purchased from the Peptide Institute, Inc. (Osaka, Japan). TRAP (H-Ser-Phe-Leu-Leu-Arg-Asn-Pro-Asn-Asp-Lys-Tyr-Glu-Pro-OH trifluoroacetate salt) was purchased from Bachem Holding AG (Budendorf, Switzerland). Phospho-specific Akt (Thr-308) antibodies and Akt antibodies were purchased from Cell Signaling Technology, Inc. (Beverly, MA, USA). Phospho-specific HSP27 (Ser-78) antibodies and the phosphorylated HSP27 (Ser-78) ELISA kit were purchased from Enzo Life Science, Inc. (Plymouth Meeting, PA, USA). HSP27 antibodies were purchased from Santa Cruz Biotechnology, Inc. (Santa Cruz, CA, USA). The PDGF-AB ELISA kit was purchased from R&D Systems, Inc. (Minneapolis, MN, USA). The other materials and chemicals were obtained from commercial sources. Aβ peptide was dissolved in dimethyl sulfoxide. The maximum concentration of dimethyl sulfoxide was 0.35%, which had no influence on platelet aggregation, protein detection by a Western blot analysis, or ELISA for PDGF-AB and phosphorylated HSP27.

### 4.2. Subjects

The inclusion criteria for the study were the presence of type 2 DM according to the criteria of the World Health Organization. We excluded the patients who were complicated with a malignancy, infectious diseases including hepatitis B and hepatitis C, or autoimmune disorders. All participants were advised to avoid sleep deprivation or blood donation. The study was approved by the committee of the conduct of human research at the National Center for Geriatrics and Gerontology (Obu, Japan), and at Gifu University Graduate School of Medicine (Gifu, Japan). Written informed consent was obtained from all of the patients and healthy donors. The subjects are patients who were hospitalized or attended National Center for Geriatrics and Gerontology between April 2019 and November 2021. This study is a prospective study.

### 4.3. Blood Sampling

Blood samples were collected from both DM patients and healthy donors. Ten ml of blood was drawn from the vein between 8:00 and 9:00 after at least 15 min of bed rest to preserve steady state conditions. A 1/10 volume of 3.8% sodium citrate was added to the blood immediately as an anticoagulant. Platelet-rich plasma (PRP) was obtained by centrifuging the samples at 155× *g* for 12 min at room temperature. Platelet-poor plasma (PPP) was obtained from the residual blood by centrifugation at 1400× *g* for 5 min.

### 4.4. Platelet Aggregation

Platelet aggregation was measured using an aggregometer (PA-200 apparatus; Kowa Co., Ltd., Tokyo, Japan) with a laser scattering system as described previously [36,37], which can detect light transmittance and the size of platelet aggregates based on particles counting (small, 9–25 μm; medium, 25–50 μm; and large, 50–70 μm). PRP was pretreated at room temperature with 10 μM of Aβ (1–40) for 15 min. After pretreatment, PRP was preincubated for 1 min at 37 °C with stirring at 800 rpm. PRP was stimulated with 8–15 μM of TRAP or vehicle, and platelet aggregation was monitored for 4 min. The dose of TRAP of the agonists was adjusted individually to achieve a percentage transmittance of >70%. The percentage of isolated PRP was recorded as 0% and that of appropriate PPP (blank) was recorded as 100%. Adjustment of PRP for platelet count does not provide any advantage and is not necessary when using light transmittance aggregometry [38]. Since an aggregometer with laser scattering that was based on light transmittance aggregometry was used, the process of PRP adjustment in the platelet count was skipped to avoid unnecessary use of time.

### 4.5. Protein Preparation after Stimulation

After stimulation, platelet aggregation was terminated by adding an ice-cold EDTA solution (10 mM). The conditioned plasma was collected and centrifuged at 10,000× *g* at 4 °C for 2 min. The supernatant was collected for each ELISA and stored at −30 °C. The pellet was washed twice with phosphate-buffered saline (PBS) and then lysed immediately by boiling in a lysis buffer containing 62.5 mM Tris-HCl (pH 6.8), 2% sodium dodecyl sulfate (SDS), 50 mM dithiothreitol, and 10% glycerol for Western blot analysis.

### 4.6. Western Blot Analysis

Western blot analysis was performed as described previously [39]. In brief, SDS-polyacrylamide gel electrophoresis (PAGE) was performed by the method described by Laemmli [40] in a 10% or 12.5% polyacrylamide gel. The proteins fractioned in the gels were transferred onto polyvinylidene fluoride (PVDF) membranes, which were then blocked with 5% fat-free dried milk in PBS with 0.1% Tween-20 (PBS-T; 10 mM Na2HPO4, 1.8 mM KH2PO4, pH 7.4, 137 mM NaCl, 2.7 mM KCL, and 0.1% Tween-20) for 2 h before incubation with phospho-specific Akt antibodies, Akt antibodies, phospho-specific HSP27 antibodies, or HSP27 antibodies as primary antibodies. Peroxidase-labeled anti-rabbit IgG antibodies or anti-goat IgG antibodies were used as secondary antibodies. The primary and secondary antibodies were diluted to their optimal concentrations with 5% fat-free dried milk in PBS-T. The peroxidase activity on the PVDF membrane was visualized on an X-ray film using an ECL Western blotting detection system (Cytiva) according to the manufacturer’s protocol.

### 4.7. ELISA for PDGF-AB and Phosphorylated HSP27

The levels of PDGF-AB and phosphorylated HSP27 in the supernatant of the conditioned mixture after platelet aggregation were determined using ELISA kits for PDGF-AB and phosphorylated HSP27, respectively, according to the manufacturer’s instructions.

### 4.8. Evaluation of WMH and Brain Atrophy

All participants underwent T1- and T2-weighted and FLAIR MR imaging with 1.5T MR scanners (Siemens Avanto, Munich, Germany; or Philips Ingenia, Eindhoven, The Netherlands). WMH and brain atrophy were analyzed by an automatic segmentation application (SNIPER, Software for Neuro-Image Processing in Experimental Research: Department of Radiology, Leiden University Medical Center, The Netherlands) [41]. Global brain atrophy was calculated as the parenchyma (PAR) volume, which is determined by subtracting the cerebrospinal fluid volume from the intracranial (IC) volume. The PAR volume corresponds to the sum of the total gray and white matter volumes. WMH and brain atrophy parameters were divided by the IC volume to adjust for the size of each patient’s brain. Further details regarding the MR scanning protocol and the automatic segmentation application are provided elsewhere [25,41,42].

### 4.9. Statistical Analysis

For the statistical significance of the difference between the two groups and the correlation between two variables, the paired *t*-test and linear regression analysis were adopted, respectively, using SPSS version 19.0 (IBM Japan Ltd., Tokyo, Japan) as the software. A probability of less than 5% was considered to be statistically significant.

## 5. Conclusions

The present study indicates that the reactivity of TRAP-stimulated platelet activation to Aβ individually differs in DM patients, and the anti-suppressive feature of platelet activation to Aβ might be protective against brain atrophy.

## Figures and Tables

**Table 1:** Characteristics of the study subjects.

Parameters	Median (IQR)
Total number (F/M)	96 (51/45)
Age (years)	76 (72–80)
DM durations (years)	12.5 (5.2–20.0)
Height (cm)	155.9 (150.0–164.3)
Weight (kg)	57.5 (53.0–64.3)
BMI	23.7 (21.7–25.7)
sBP (mmHg)	124 (112–133)
dBP (mmHg)	67 (59–77)
HbA1c (%)	8.5 (7.4–9.8)
Glu (mg/dL)	130 (106.5–162.0)
TC (mg/dL)	185 (160.7–208.2)
TG (mg/dL)	115 (91.5–142.5)
HDL (mg/dL)	50 (39.2–61.7)
Plt (×10^4^/μL)	20.0 (17.4–24.0)
WMH/IC (×10^−2^)	0.304 (0.112–0.758)
PAR/IC	0.802 (0.749–0.830)

F, female; M, male; DM, diabetes mellitus; BMI, body mass index; sBP, systolic blood pressure; dBP, diastolic blood pressure; HbA1c, hemoglobin A1c; Glu, plasma glucose; TC, total cholesterol; TG, triglyceride; HDL, high-density lipoprotein; Plt, platelet counts; WMH, white matter hyperintensity; IC, intracranial; PAR, parenchyma. The data are presented as the median (interquartile range).
